# Translation and validation of the Dutch version of the health professional education in patient safety survey amongst nursing students in Belgium: A psychometric analysis

**DOI:** 10.1371/journal.pone.0247869

**Published:** 2021-03-03

**Authors:** Jochen Bergs, Katrien Peeters, Isabel Kortleven, Sarah Creemers, Dorien Ulenaers, Melissa Desmedt, Ward Schrooten

**Affiliations:** 1 Faculty of Medicine and Life Sciences, Hasselt University, Hasselt, Belgium; 2 School of Educational Studies, Hasselt University, Hasselt, Belgium; 3 Department of Healthcare, PXL University of Applied Sciences and Arts, Hasselt, Belgium; 4 Department of Healthcare, UC Leuven-Limburg, Genk, Belgium; 5 Faculty of Business Economics, Hasselt University, Hasselt, Belgium; University of Antwerp, BELGIUM

## Abstract

**Objectives:**

Evaluate the psychometric properties of the Dutch version of the Health Professional Education in Patient Safety Survey (H-PEPSS_Dutch_), an instrument used to assess self-efficacy regarding patient safety competence.

**Methods:**

The H-PEPSS_Dutch_ was administered to 610 students in two Belgian nursing schools. We used confirmatory factor analysis, for both classroom and clinical learning, to examine the psychometric properties.

**Results:**

The analysis of construct validity showed a good fit to the hypothesised models. Cronbach’s alpha values ranged from 0.70 to 0.87 for classroom learning and from 0.56 to 0.86 for clinical learning, indicating good reliability. Differentiating between the H-PEPSS constructs in the clinical setting showed to be complicated; hence, discriminant validity was not supported for all dimensions.

**Conclusions:**

Overall, this provides us with a reliable instrument to measure self-reported patient safety competence among nursing students. Further research is needed to validate the H-PEPSS as a longitudinal monitoring tool and as a pre-and-post measurement on the impact of interventions related to patient safety in the nursing curricula.

## Introduction

Patient safety incidents are still reaching patients, with a profound impact on their lives. Overall, the available evidence suggests that 15% of hospital expenditures and activity can be attributed to treating safety failures [1]. In an attempt to decrease preventable healthcare-related harm, improvement efforts have mainly focused on system responses (*e*.*g*., guidelines, incident reporting, education and training) and structural changes (*e*.*g*., reorganising, accreditation) [2]. However, the effects of these strategies appear to be limited, as no significant improvement can be found over time [3–5]. Hence, a more comprehensive framework is needed to comprise patient safety to its full extent. An evidence-based framework of contributing factors to patient safety incidents identified 20 key domains [6]. This framework shows that it is essential to not only focus on the tangible aspects of patient safety, yet more importantly recognise the importance of socio-cultural elements (*i*.*e*., a culture of safety, communication, team functioning) as critical causal factors [7].

Improving patient safety requires (future) healthcare professionals to be educated on these socio-cultural aspects of patient safety. The World Health Organisation developed the *Multi-professional Patient Safety Curriculum Guide* to provide universities and schools with the requirements and tools for incorporating patient safety in education [8]. In this curriculum guide, both clinical and socio-cultural aspects of patient safety are addressed; the latter in correspondence with the socio-cultural competencies determined in the safety competencies project initiated by the Canadian Patient Safety Institute (CPSI).

When assessing patient safety competencies, there is a need for validated instruments. Ginsburg *et al*. developed the *Health Professional Education in Patient Safety Survey* (H-PEPSS), an instrument designed to reflect on the six socio-cultural safety competencies of the CPSI [9]. Since the introduction in 2012, psychometric properties of the H-PEPSS have been tested by the original developers, and several translations of the original questionnaire have been published [10–15]. In the Belgian context, a Dutch version was needed to evaluate patient safety competencies among nursing students. Therefore, this study aimed to translate the H-PEPSS into Dutch and retest its psychometric properties.

## Methods

This paper reports on the translation and psychometric analysis of the Dutch version of the *Health Professional Education in Patient Safety Survey* (H-PEPSS_Dutch_). We used published recommendations and guidelines during the preparation and reporting of this study [16, 17].

### Design and setting

We conducted a psychometric evaluation study of the H-PEPSS_Dutch_ using survey data from a cross-sectional sample of Bachelor of Nursing students from two nursing schools in the Belgian province of Limburg. The study was conducted in May-June 2017.

In Belgium, nursing education is provided at two levels: at European Qualifications Framework (EQF) level 5 (HBO-5) and EQF level 6 (Bachelor of Nursing). Level 5 nursing is organised as a three-year program (180 ECTS). The Bachelor of Nursing curriculum covers a four-year program (240 ECTS). Both consist of a combination of classroom teaching and clinical placements in all years. Both educational programs comprise 2,300 hours of clinical placement and give access to the same title of “nurse responsible for general care”, as defined in the European Directive 2013/55/EC. Supervision and coaching during clinical placement are organised differently within these programs. Within the level 5 program, a supervisor, associated with the nursing school, works alongside the student on a weekly basis. Within the bachelor’s program, the supervisor rather acts as a coach, guiding the students learning trajectory. Additional practical supervision during placement is carried out by nurses working in the clinical sites (preceptors).

In this study, all participants were students in the Bachelor of Nursing curriculum. However, the curriculum was reformed in academic year 2016–2017, from a 3 to a 4-year program, in order to adhere to the requirements of European Directive 2013/55/EC. First-year students began the renewed four-year program, while students of the second and third year were still enrolled in the three-year program. Both programs are organised in the same way in terms of teaching methodology—using lectures, tutorials, response lectures, skills training, and simulation education. They also provide similar content, yet in comparison to HBO-5, place a stronger emphasis on clinical leadership, clinical reasoning, and evidence-based nursing. Clinical placements occur in various settings (*e*.*g*., acute, long-term, and community care). An important distinction between the former three-year and the current four-year program can be found in clinical placement duration. The amount of placement hours had to be increased significantly to comply with the European directive. To maintain the theoretical content, the programme was extended by one academic year (60 ECTS).

Besides, there is also a difference in the educational approach between the two schools; one uses problem-based learning; the other uses assignment-based learning.

### Sample and data collection

Based on the recommendations of Hair *et al*., it is advised to use at least ten times the number of subjects for each item on the scale [18]. As the H-PEPSS has 16 items, the calculated sample size for this study had to be, at least, 160 individuals.

All students were eligible to participate. They were invited to participate in this study by e-mail and a verbal invitation during one of the patient safety related courses by one of the teachers involved in this study (IK & KP). Two reminders were sent out one and two weeks after the initial invitation. We undertook several steps to mitigate the risk of common method bias, both ex-ante remedies as well as statistical controls after the questionnaires were used (*e*.*g*., respondents were assured of confidentiality and it was made clear that there were no ’right’ or ’wrong’ answers) [19].

### The original H-PEPSS

The H-PEPSS is designed to measure health professionals self-reported patient safety competence [9]. The dimensions included in the H-PEPSS were theoretically derived to reflect the six domains of the CPSI safety competencies framework and focus primarily on the socio-cultural aspects of patient safety [9].

The first section of the questionnaire (20 items) is focused on learning about specific patient safety content areas. To help respondents differentiate between clinical and socio-cultural aspects of patient safety, the H-PEPSS starts by asking about confidence in the knowledge of four clinical aspects of patient safety (*i*.*e*., hand hygiene, infection control, safe medication practices, and safe clinical practice in general) [9]. This helps to focus on the other 16 items, which are intended to reflect the six domains of the CPSI safety competencies framework: *Working in teams* (three items), *Communicating effectively* (three items), *Managing risks* (three items), *Understanding human and environmental factors* (two items), *Recognising and responding to adverse events* (two items), and *Culture of safety* (three items). The second section (seven items) is focused on how broader patient safety issues are addressed in health professional education and aims to gain an overall understanding of students’ perceptions regarding their patient safety education. The third section (four items) askes how able and comfortable they feel speaking up about patient safety. In this study we focus on the 16 items measuring the six dimensions of the CPSI safety competencies framework.

All items are answered using a five-point Likert scale ranging from 1 (disagree) to 5 (agree) and include a ’don’t know’ option. For each item, respondents are asked to respond to their confidence in what they learnt in the classroom setting and during clinical placements. This is done separately given the different educational experiences, and often inconsistency exists in how patient safety issues are conveyed in the classroom and clinical setting [9]. Dimensional scores are calculated as the mean score of the items in each dimension. This is done separately for each learning setting; for each dimension, you will have a rating for confidence in learning based on education provided in the classroom and a separate score based on learning during clinical placements. For more detailed information, we refer to the H-PEPSS website: http://www.yorku.ca/patientsafety/H-PEPSS/.

### Translation and cultural adaptation

When introducing a foreign language questionnaire, potential semantic and cultural differences need to be considered [20]. To determine semantic equivalence, the H-PEPSS was translated and adapted from English into Dutch following the WHO guidelines [21]. The process included translation, expert panel discussion, back translation, pilot test, and cognitive interviews.

After obtaining written approval of the original authors, the questionnaire was individually translated by four nursing educators and two patient safety researchers (native Dutch, fluent in English). In the next step, the six resulting documents were presented to a subject matter expert group; consisting of three patient safety researchers, three educational experts, and one English teacher-translator with English as a native language. This expert group analysed and consolidated, by consensus, the six translations obtained by selecting the best wording and expressions. After receiving a single translation, clarity and appropriateness of wording and each item’s meaning in Belgium’s cultural setting were analysed. Back translation was performed to assure alignment with the original intention of the questions.

Cognitive semi-structured interviews were conducted with ten students to determine whether the H-PEPSS_Dutch_ was understandable and meaningful. The emphasis of the interviews was also to determine whether essential concepts were understood correctly; minor adjustments in wording were made based on the students’ feedback. The final version of the H-PEPSS_Dutch_ is included in the S1 File.

### Statistical analysis

Respondent characteristics were summarised using proportions for discrete variables, average scores for ordered categorical variables, and means (*M*) with standard deviations (*SD*) and ranges for continuous variables. As the domains included in the H-PEPSS were theoretically derived, confirmatory factor analysis (CFA) was deemed appropriate.

### Pre-analysis

To check whether the data were appropriate for confirmatory factor analysis, the Kaiser-Meyer-Olkin measure of sampling adequacy (> 0.70) and Bartlett’s test of sphericity (*P*<0.01) was performed [18].

### Confirmatory factor analysis

A first-order confirmatory factor analysis (CFA) was performed to evaluate the congruence of the factorial structure emerging in the validation process of the original scale in the H-PEPSS_Dutch_. This was done for both classroom and clinical learning. Diagonally weighted least squares (DWLS) was used as the extraction method, as data did not meet the assumption of multivariate normality and are not continuous. The following fit indices were used to evaluate model fit:

- The chi-square (χ^2^) test; non-significant χ^2^ values indicate an acceptable fit of the model [22].
- The root mean square error of approximation (RMSEA); values of ≤ .05 and ≤ .01 indicate, respectively, good and excellent fit. For a model with 16 measures and a sample size of 484, a threshold of < 0.07 is recommended [18]; The RMSEA can also be evaluated in terms of probability, with a *P*-value less of.05.
- The normed χ^2^; values of <2.0 suggest good fit;
- The standardised root mean square residual (SRMR); values of ≤ .08 demonstrate a good fit ≤ .05 shows excellent fit;
- The comparative fit index (CFI); values of ≥ .90 indicate an acceptable fit and ≥ .95 indicate an excellent fit.
- The Tucker Lewis index (TLI); the thresholds are the same as CFI [18, 23, 24].

### Content validity

Correspondence between the individual items and the concepts was assessed during translation and pretest. No significant alternations were suggested by the expert team, nor by the pretest sample.

### Reliability

Reliability was tested using the following indices: (a) the Cronbach’s *α*, (b) the Composite Reliability (CR) that must be ≥ .70 for satisfactory reliability. Reliability indices between 0.6 and 0.7 may be acceptable, providing those other indicators of a model’s construct validity are good [18].

### Construct validity

The convergent and discriminant validity of the models were tested. For convergent validity, the factor loading is one important consideration. At a minimum, all factor loadings should be statistically significant. Because a significant loading could still be fairly weak in strength, a good rule of thumb is that standardised loading estimates should be 0.5 or higher, and ideally 0.7 or higher. To measure the covariance between the constructs, we created a construct correlation matrix, expressed as standardised correlations. Further, the Average Variance Extracted (AVE), that must be ≥ 0.50, was calculated [18]. For discriminant validity, the Maximum Shared Squared Variance (MSV) and the Average Shared Squared Variance (ASV) were measured; both must be lower than the AVE [18].

All analyses were performed using R: A Language and Environment for Statistical Computing version 3.2.2., the Lavaan package was used to perform CFA [25, 26].

### Ethical considerations

A full proposal outlining all study methods and stages was reviewed by the Institutional Review Boards (IRB) of both nursing schools (IRB of UCLL Limburg and IRB PXL university of applied sciences and arts), who granted permission for the study to proceed. Students received a full informed consent form (ICF) with the invitation e-mail. The ICF contained all information about the study objectives, their rights as participants, and how the data would be stored and processed. When they visited the survey website a summary of the ICF was presented, they could only proceed to the questionnaire after confirming their consent. The anonymity of the participants was guaranteed at all times. No incentives were offered to participants. We informed the participants that the collected information would be kept confidential and that the questionnaire was anonymous. No incentives were provided for completing the survey.

## Results

In total, 484 valid questionnaires were returned from 610 students, representing a response rate of 79%. Most of the respondents were female (n = 397, 82.02%). The median age was 21 years, ranging from 18 to 50 years. The majority of respondents were 2^nd^ year bachelor students (n = 176, 36.36%), followed by 1^st^ (n = 166, 34.3%) and 3^rd^ year students (n = 142, 29.34%).

### Classroom learning

The goodness-of-fit statistics, as presented in Table 1, are all within the predetermined thresholds, therefore provide sufficient evidence to assume good model fit [18].

**Table 1:** Goodness-of-fit indices.

Measure	Threshold	Classroom learning	Clinical learning
χ^2^ (df), P-value	-	97.77(89), 0.25	83.42(89), 0.65
RMSEA, [90%CI]	<0.07	0.01, [0.00–0.03]	0.01, [0.00–0.02]
SRMR	≤0.08	0.03	0.03
Normed χ^2^	<2.0	1.1	0.94
CFI	>0.92*	0.99	0.99
TLI	>0.92*	0.99	0.99

(df) = degrees of freedom; RMSEA = root mean square error of approximation; 90%CI = 90% confidence interval; SRMR = standardized root mean square residual, CFI = comparative fit index; TLI = Tucker-Lewis index;

*for a model of this complexity and sample size.

### Convergent validity

**Fig 1:**
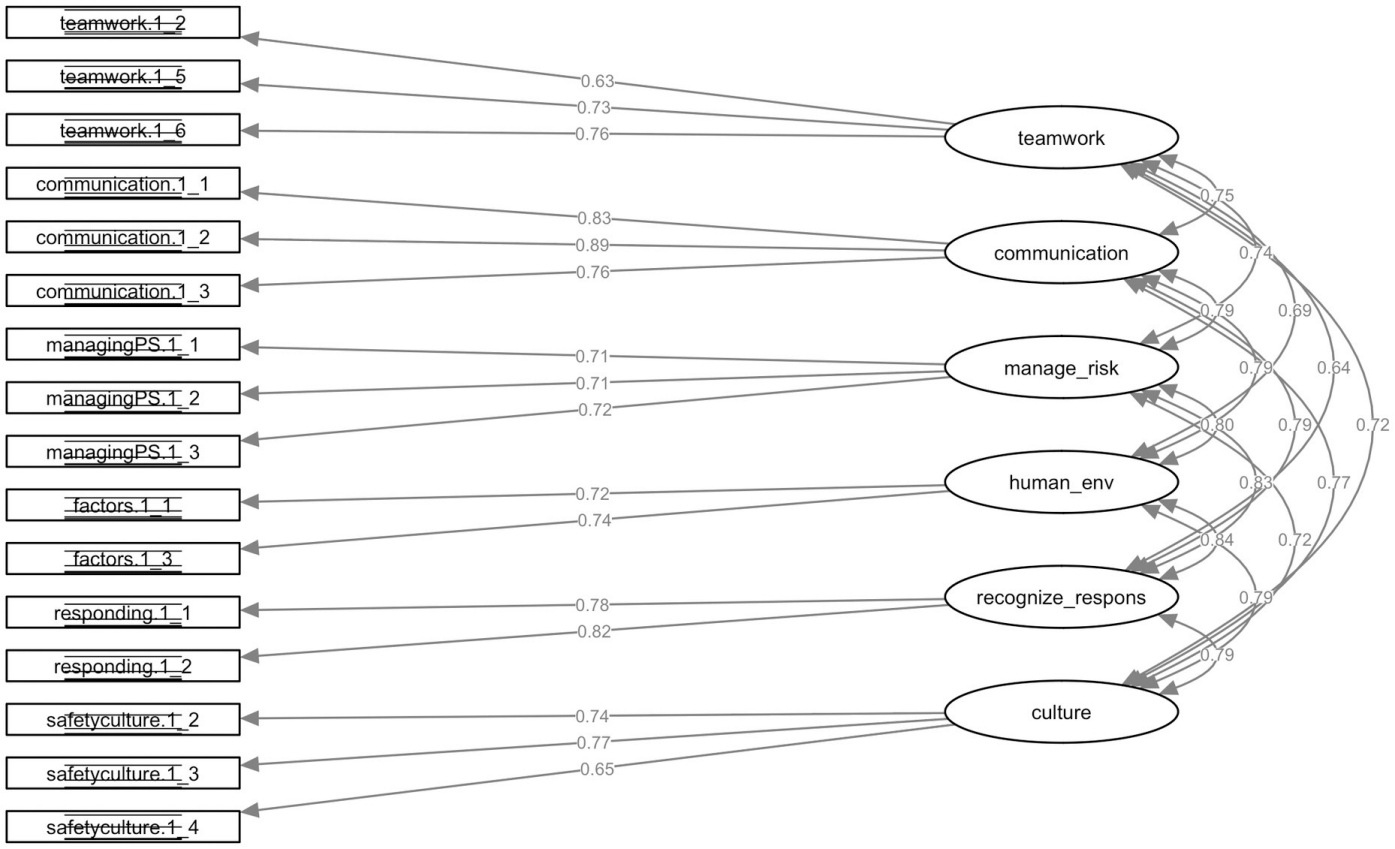
Confirmatory factor analysis of the H-PEPSS model for classroom learning. This figure shows the standardised path coefficients (factor loadings) and factor covariances of the six-dimensions of the H-PEPSS.

All loadings in the classroom learning model are highly significant, as required for convergent validity. According to the first-order constructs, the standardised parameter loadings ranged from 0.63 to 0.76 for *Work in teams for effective patient safety*, *Communicate effectively for patient safety* ranged from 0.76 to 0.89, *Manage safety risks* ranged from 0.71 to 0.72, *Optimise human and environmental factors* ranged from 0.72 to 0.74, *Recognise*, *respond to and disclose adverse events* ranged from 0.78 to 0.82, and *Contribute to a culture of patient safety* ranged from 0.65 to 0.77, which are all above the 0.50 cutoff value (Fig 1) [18]. The AVE’s range from 50% for *Work in teams for effective patient safety* to 69% for *Communicate effectively for patient safety*. All AVE’s are at least 50%, suggesting adequate convergence. Composite reliabilities are above 0.70 for most factors, except for *Work in teams for effective patient safety* (0.69) and *Understanding human and environmental factors* (0.63). Cronbach’s *α* values for all factors are greater than the 0.7 cutoff. Taken together, the evidence supports the convergent validity of the measurement model. A detailed overview of the validity indices is shown in Table 2.

**Table 2:** Construct validity—Classroom learning.

	Factor 1	Factor 2	Factor 3	Factor 4	Factor 5	Factor 6
SPM	0.63–0.76	0.76–0.89	0.71–0.72	0.72–0.74	0.78–0.82	0.65–0.77
AVE	0.50	0.69	0.51	0.53	0.65	0.52
Cronbach’s *α*	0.74	0.87	0.76	0.70	0.78	0.76
CR	0.69	0.82	0.70	0.63	0.72	0.72
MSV	0.57	0.63	0.69	0.70	0.70	0.63
ASV	0.42	0.51	0.50	0.51	0.51	0.48

SPM = standardized parameter loadings; AVE = average variance extracted; alpha = Cronbach’s alpha; CR = Composite Reliability; MSV = Maximum Shared Squared Variance; ASV = Average Shared Squared Variance.

Factors: *Working in teams* (factor 1), *Communicating effectively* (factor 2), *Managing risks* (factor 3), *Understanding human and environmental factors* (factor 4), *Recognising and responding to adverse events* (factor 5), and *Culture of safety* (factor 6).

### Discriminant validity

The data in Table 3 shows that not all AVE estimates are greater than the corresponding squared interconstruct correlations estimates. Therefore, this test indicates that their discriminant validity could not be established for the classroom learning model.

**Table 3:** Construct correlation matrix (standardized)—Classroom learning.

	Factor 1	Factor 2	Factor 3	Factor 4	Factor 5	Factor 6
*Working in teams* (factor 1)	1.00	.57	.55	.48	.41	.51
*Communicating effectively* (factor 2)	.75***	1.00	.62	.63	.63	.59
*Managing risks* (factor 3)	.74***	.79***	1.00	.64	.69	.51
*Understanding human and environmental factors* (factor 4)	.69***	.79***	.80***	1.00	.70	.63
*Recognising and responding to adverse events* (factor 5)	.64***	.79***	.83***	.84***	1.00	.63
*Culture of safety* (factor 6)	.72***	.77***	.72***	.79***	.79***	1.00

Significance Level:

* = .05,

** = .01,

*** = .001.

Note: Values below the diagonal are correlation estimates among constructs, diagonal elements are construct variances, and values above the diagonal are squared correlations.

Factors: *Working in teams* (factor 1), *Communicating effectively* (factor 2), *Managing risks* (factor 3), *Understanding human and environmental factors* (factor 4), *Recognising and responding to adverse events* (factor 5), and *Culture of safety* (factor 6).

### Clinical setting learning

The goodness-of-fit statistics for the learning in the clinical setting, as presented in Table 1, are all within the predetermined thresholds, therefore provide sufficient evidence to assume good model fit [18].

### Convergent validity

**Fig 2:**
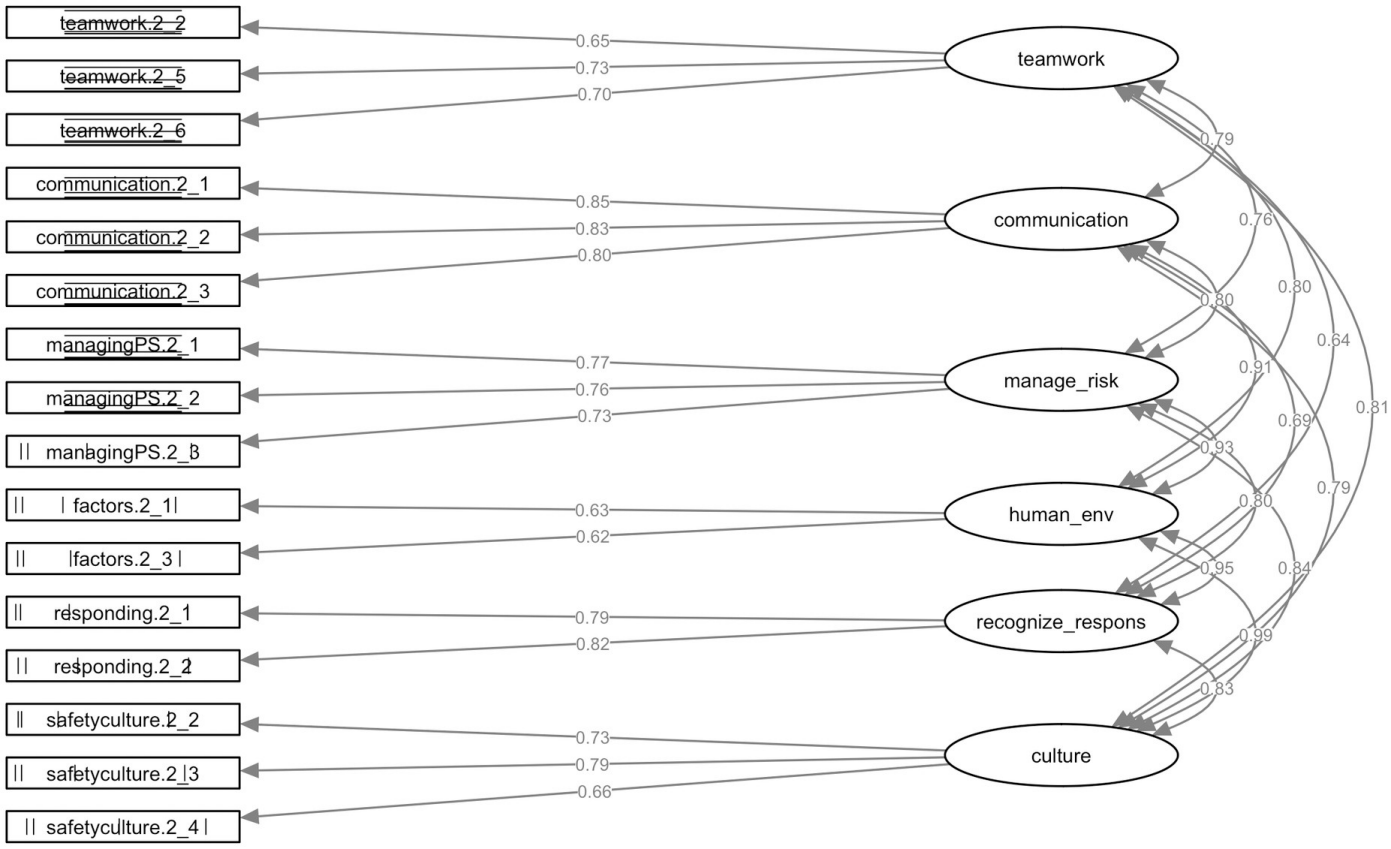
Confirmatory factor analysis of the H-PEPSS model for clinical learning. This figure shows the standardised path coefficients (factor loadings) and factor covariances of the six-dimensions of the H-PEPSS.

All loadings in the clinical setting learning model are highly significant. The standardised parameter loadings range from 0.65–0.73 for *Work in teams for effective patient safety*, *Communicate effectively for patient safety* ranged from 0.79–0.85, *Manage safety risks* ranged from 0.73 to 0.77, *Optimise human and environmental factors* ranged from 0.62 to 0.63, *Recognise*, *respond to and disclose adverse events* ranged from 0.79 to 0.81, and *Contribute to a culture of patient safety* ranged from 0.66 to 0.79, which are all above the 0.50 cutoff value (Fig 2) [18]. Further, the AVE’s range from 39% for *Understanding human and environmental factors* to 68% for *Communicate effectively for patient safety*. As not all AVE’s exceed the threshold, the data shows weak convergence for two factors: *Working in teams (48%)* and *Understanding human and environmental factors (39%)*.

Composite reliabilities are above 0.70, except for *Work in teams for effective patient safety* (0.67) and *Understanding human and environmental factors* (0.50). Cronbach’s *α* values are higher than the 0.7 cutoff, except for the factor *Understanding human and environmental factors*. The AVE estimates and construct reliabilities are shown in Table 4.

**Table 4:** Construct validity—Clinical learning.

	Factor 1	Factor 2	Factor 3	Factor 4	Factor 5	Factor 6
SPM	0.65–0.73	0.79–0.85	0.73–0.77	0.62–0.63	0.79–0.81	0.66–0.79
AVE	0.48	0.68	0.57	0.39	0.64	0.53
Cronbach’s *α*	0.73	0.86	0.79	0.56	0.78	0.76
CR	0.67	0.81	0.74	0.50	0.72	0.72
MSV	0.66	0.83	0.86	0.97	0.90	0.97
ASV	0.49	0.53	0.57	0.70	0.52	0.61

SPM = standardized parameter loadings; AVE = average variance extracted; alpha = Cronbach’s alpha; CR = Composite Reliability; MSV = Maximum Shared Squared Variance; ASV = Average Shared Squared Variance.

Factors: *Working in teams* (factor 1), *Communicating effectively* (factor 2), *Managing risks* (factor 3), *Understanding human and environmental factors* (factor 4), *Recognising and responding to adverse events* (factor 5), and *Culture of safety* (factor 6).

### Discriminant validity

Strong correlations between factors exist. Hence, not all AVE estimates are greater than the corresponding squared interconstruct correlations estimates (See Table 5). Therefore, this test indicates that discriminant validity could not be established for the classroom learning model.

**Table 5:** Construct correlation matrix (standardised)—Clinical learning.

	Factor 1	Factor 2	Factor 3	Factor 4	Factor 5	Factor 6
*Working in teams* (factor 1)	1.00	.62	.58	.65	.41	.66
*Communicating effectively* (factor 2)	.79***	1.00	.65	.83	.48	.63
*Managing risks* (factor 3)	.76***	.80***	1.00	.86	.64	.70
*Understanding human and environmental factors* (factor 4)	.81***	.91***	.93***	1.00	.90	.97
*Recognising and responding to adverse events* (factor 5)	.64***	.69***	.80***	.95***	1.00	.68
*Culture of safety* (factor 6)	.81***	.79***	.84***	.99***	.82***	1.00

Significance Level:

* = .05,

** = .01,

*** = .001.

Note: Values below the diagonal are correlation estimates among constructs, diagonal elements are construct variances, and values above the diagonal are squared correlations.

Factors: *Working in teams* (factor 1), *Communicating effectively* (factor 2), *Managing risks* (factor 3), *Understanding human and environmental factors* (factor 4), *Recognising and responding to adverse events* (factor 5), and *Culture of safety* (factor 6).

## Discussion

This paper presents the translation process, pilot testing, and psychometric analysis of the Dutch version of the H-PEPSS, known as H-PEPSS_Dutch_. The analysis of construct validity, based on the CFA and goodness-of-fit indices, showed a good fit to the hypothesised models. Our results showed satisfactory factor loadings, suggesting that the H-PEPSS_Dutch_ reflects the constructs of the underlying six domains of the CPSI safety competencies framework. The internal consistency for the six constructs reflected by H-PEPSS_Dutch_ in terms of Cronbach’s alpha values ranged from 0.70 to 0.87 for classroom learning and from 0.56 to 0.86 for clinical learning, indicating good reliability.

In our study, the results for factor *Understanding human and environmental factors* do not meet the predefined criteria for confirming construct validity. Before assuming this to be a negative evaluation of the value and usefulness of the H-PEPSS, we would like to put these findings into context. As the standards for what makes a “good” alpha coefficient are entirely arbitrary and depend on your theoretical knowledge of the scale in question. First, the factor Understanding human and environmental factors consists of two items aimed at two different factors influencing patient safety, *i*.*e*., human factors and environmental factors. Despite being a seemingly logical (theoretical) combination of factors affecting patient safety, unidimensionality is arguable. Learning about these aspects will most likely take place at separate occasions, especially during clinical placement. As exposure to these two types of factors most likely occurs asynchronously, this translates into varying degrees of self-efficacy. Hence, resulting in lower Cronbach’s alpha values. Second, building in the first argument, one can even question the added value of psychometric analysis for this variable. The information we are interested in is not characterised as a latent variable—which can only be measured by means of n defining items. The information that this dimension reflects is directly observable. The fact that this dimension consists of two distinct items is to be found in the theoretical foundation of the H-PEPSS. From this point of view we should be careful not drifting down into a mathematical discussion while there are sound theoretical arguments for the structure of the questionnaire. Last, it is difficult to compare these values directly across studies. Despite this, the factor Understanding human and environmental factors often performs as the least coherent factor. Combines, these findings are in line with previously published results [9–13].

This paper is among the first to report on the convergent and discriminant validity of the HPEPSS. Our findings showed that discriminant validity was not supported for all dimensions. To establish discriminant validity, you need to show that measures which should not be related are in reality not related. From a theoretical point of view, there is indeed a difference between the socio-cultural constructs used in the H-PEPSS. Some of these constructs (*e*.*g*., communication, teamwork, and safety culture) are also separately used in broader research on patient safety. For instance, communication and teamwork are frequently used aspects in the various definitions and instruments for safety culture. However, contrary to the theoretical differences, in practice, there is a strong correlation and mutual influence between them. The interdependence between these constructs is also acknowledged in the literature [27]. Furthermore, controversies in the definitions and dimensions of safety culture in healthcare are still present in today’s literature. Evidence of the adequacy of the psychometric development of safety culture questionnaires is still limited, which also reflects upon the H-PEPSS as it intends to measure students’ self-efficacy regarding the socio-cultural aspects of patient safety [28].

The results of this study must be interpreted within the frame of the theoretical underpinning used to inform the development of the H-PEPSS [9]. One must keep in mind that the H-PEPSS intends to measure students’ self-efficacy regarding patient safety competencies. So, it does not intend to measure the presence or quality of these constructs in the learner’s environment. Assessing how confident you are about what you have learned differs from determining the quality of the same constructs in a specific context. It is not inconceivable that, when learning about these constructs, boundaries between socio-cultural concepts are more blurred than when assessing their quality. For instance, communication and teamwork are often included in the same course. Therefore, it may not be unexpected that the difference between these constructs is vague, especially when reflecting on learning about these aspects in clinical practise where the boundaries between these constructs are even more ambiguous than in theoretical lessons and practical exercises. The lack of evidence for discriminant validity in this study must, therefore, lead to an in-depth analysis of students understanding about the differences and their ability to differentiate between them.

Patient safety, and its related competencies, are often presented cross-curricular. As a result, learning takes place within various aspects of the programme (*e*.*g*., Learning during clinical placements). This exposure has a big, if not the biggest, impact on learning about patient safety. Reflection on learning about patient safety in the clinical setting is not based on a single experience, as most students do several internships per year. Moreover, scoring is probably affected most by their most positive or negative experience. Besides centric measures, analysing variation in scoring could be a useful application in monitoring patient safety competencies among healthcare professional students.

The translation methodology used in this study has been recommended as a reliable method for translating questionnaires for research utilisation. However, there is no golden standard for questionnaire translation and adaptation. Hence, the use of multiple methods is commonly recommended [21]. For instance, we included experts with distinct backgrounds in the translation process to secure the cross-cultural validity of H-PEPSS_Dutch_. Besides, the cultural adaptation was not considered as being a separate step; it was performed throughout the entire translation process. The changes in wording that were made during translation and adaptation are considered to be carefully and methodically performed and conducted with sensitivity to the original purpose and theoretical foundation of the H-PEPSS. Therefore, the core of the instrument should remain the same.

### Implications for practice and research

Results of this instrument can provide us with insight in perceived patient safety competencies throughout the curriculum, and function as a tool to assess and improve patient safety education. Self-reported competence is likely to be boosted when patient safety knowledge, skills, and attitudes are integrated consistently across all learning settings and progressively evolve throughout the curriculum [9, 29]. Knowing the impact of learning about patient safety in the classroom versus the clinical setting can educate us about strengths and weaknesses in current curricula. Measuring perceived patient safety competence is only a first step. The ultimate goal is to make a profound and relevant impact on patient safety in clinical practice. Hence, based upon the data provided by instruments like the H-PEPSS, an adjustment in the curriculum and educational strategies must be implemented. Future research must establish the H-PEPSS as a valid longitudinal monitoring tool and as a tool for pre-and-post measurement on the impact of interventions related to the improvement of patient safety competence in the curricula. Besides, the results can also be used as an entry point for a more in-depth exploration of the meaning, acquisition, and embedding of socio-cultural competencies in healthcare education. These objectives require a qualitative approach, which, in addition to quantitative data, can provide a broader view of the constructs under study. Further research is needed to resolve the controversies in the definitions and dimensions around the concepts of safety culture and climate in healthcare.

### Limitations

When interpreting the results of this study, some limitations must be kept in mind. First, the current reformation of the curriculum, from three to four years, has led to shifting courses and topics. For instance, topics about nursing handover are now being taught as early as the first year of the curriculum. However, this reflects the ongoing educational reform and continuing refinement of the nursing curriculum. Furthermore, when comparing nursing schools, we must take into account the fact that the curricula are not identical. In addition, the school climate and teachers’ attitude also has a formative role—*e*.*g*., a climate during simulation practice in which making mistakes is punished, contributes to the maintenance of a "shame-and-blame" culture among students. Hence, when using the results of this questionnaire to improve healthcare professional education, it is important to go beyond the analysis of the curriculum and learning methods offered, and also reflect upon the institutions’ vision on the underlying socio-cultural dimensions and their representation by its staff members.

## Conclusions

The results of this study confirm the construct validity and reliability of the H-PEPSS_Dutch_ in a sample of Belgian nursing students. Our results show some reservations regarding the divergent validity of the H-PEPSS for learning in the clinical setting. The distinction between some factors seems to be difficult, especially when reflecting upon self-efficacy regarding the socio-cultural aspects of patient safety in the clinical setting. Further research is needed to validate the H-PEPSS as a longitudinal monitoring tool and as a pre-and-post measurement on the impact of interventions related to patient safety in the nursing curricula.

## Supporting information

S1 File The H-PEPSS_Dutch_. The Dutch version of the health professional education in patient safety survey (H-PEPSS_DUTCH_).
(PDF)

S2 File (CSV)
