# Supplementary material for: Translation and validation of the Dutch version of the health professional education in patient safety survey amongst nursing students in Belgium: A psychometric analysis
Source: PLoS One. 2021 Mar 3;16(3):e0247869. doi: 10.1371/journal.pone.0247869 (PMC7928461; doi:10.1371/journal.pone.0247869)
Supplement: S1 File — The Dutch version of the health professional education in patient safety survey (H-PEPSSDUTCH). (PDF) [file pone.0247869.s001.pdf]

Questionnaire

H-PEPSS$_{Dutch}$

Health Professional Education in Patient Safety Survey

UHASSELT

KNOWLEDGE IN ACTION

Questionnaire

H-PEPSS$_{Dutch}$: Dutch translation of the Health Professional Education in Patient Safety Survey

Jochen Bergs PhD, MSc, RN
Faculty of Medicine and Life Sciences,
Hasselt University,
Belgium.

Isabel Kortleven MSc, RN
Faculty of Healthcare,
PXL university college,
Belgium.

Karien Peeters MSc, RN
Faculty of Healthcare,
University college Leuven-Limburg,
Belgium.

© De auteur(s). 2017. This work is licensed under a Creative Commons Attribution 4.0 International License (CC BY 4.0), which permits others to copy and redistribute the material in any medium or format and to adapt and build upon the material for any purpose, even commercially, provided the original work is properly cited. See: https://creativecommons.org/licenses/by/4.0/

Over de H-PEPSS vragenlijst

Dit is de Nederlandse vertaling van de *Health Professional Education in Patient Safety Survey.* Toestemming voor het vertalen van de originele versie en gebruik van deze vragenlijst is bekomen bij de auteurs van het originele werk. Voor meer informatie over de originele vragenlijst verwijzen wij u naar volgende website: http://www.yorku.ca/patientsafety/H-PEPSS/
Hoewel wij u toestaan onze Nederlandstalige vertaling vrij te gebruiken raden wij u aan bij gebruik ervan de originele auteurs van de H-PEPSS te contacteren en hun toestemming voor het gebruik van deze vragenlijst te bekomen.

Binnen deze enquête bevragen we de perceptie van de studenten in gezondheidsberoepen over de manier waarop patiëntveiligheid in hun opleiding voorkomt. De H-PEPSS vragenlijst stelt vragen vraagt over klinische veiligheidsproblemen zoals handhygiëne, overdracht van patiënten en medicatie veiligheid maar ook over systeemproblemen die patiëntveiligheid beïnvloeden, zoals aspecten van de organisatie, management of de werkomgeving inclusief beleid, middelen, communicatie en andere processen.

De H-PEPSS vragenlijst omvat 38 vragen verdeeld in drie secties. De eerste sectie bevraagt hoe vertrouwd studenten zijn met de leerinhoud over 7 domeinen van patiëntveiligheid. Geef aan in welke mate jij je vertrouwd voelt met wat je geleerd hebt over elk van deze specifieke domeinen. We vragen je om na te denken over je ervaringen, enerzijds binnen de opleiding (tijdens de lessen), anderzijds binnen het werkveld (tijdens stages). Beoordeel beide settingen afzonderlijk. In sectie twee peilen de vragen naar hoe bredere kwesties inzake patiëntveiligheid in de opleiding van gezondheidszorg professionals, zowel het theoretische als het klinische onderwijs, worden besproken. Sectie drie ten slotte toetst in welke mate je je veilig voelt (comfortabel voelt) om te spreken over patiëntveiligheid.

Onder patiëntveiligheid wordt het volgende verstaan: Het streven naar vermindering en beperken van onveilige handelingen binnen de gezondheidszorg, alsmede het gebruik van de beste praktijken die blijken te leiden tot optimale patiëntenzorguitkomsten.

Uitnodiging tot deelname

Beste student,

Hartelijk dank voor de tijd die u neemt om deze vragenlijst in te vullen. Aan de hand van een aantal stellingen proberen we inzicht te krijgen in het beeld dat studenten in gezondheidsberoepen hebben over hetgeen zij leren over patiëntveiligheid tijdens hun opleiding. De resultaten van deze studie zullen gebruikt worden om de opleiding omtrent patiëntveiligheid te verbeteren. Om te kunnen achterhalen wat er leeft bij de studenten is het belangrijk dat u de vragenlijst oprecht invult en niets blanco laat. Er zijn geen juiste of foute antwoorden. Bij vragen of opmerkingen kan u steeds terecht bij een van de onderzoekers.

Nogmaals hartelijk dank voor uw deelname aan dit onderzoek.

Met vriendelijke groeten,
Het H-PEPSS$_{dutch}$ projectteam

Prof. dr. Jochen Bergs, UHasselt, jochen.bergs@uhasselt.be
drs. Dorien Ulenaers, UHasselt, dorien.ulenaers@uhasselt.be
mevr. Isabel Kortleven, PXL, isabel.kortleven@pxl.be
mevr. Katrien Peeters, UCLL, katrien.peeters@ucll.be

INSTRUCTIES:
1. Het invullen van deze enquête duurt ongeveer 12 minuten.
2. De enquête onderzoekt alleen uw opvattingen en meningen. Er zijn dus geen juiste of verkeerde antwoorden. Geef aan in welke mate u het eens bent of niet eens bent met de onderstaande stellingen. Als u niet zeker bent of u het eens bent of oneens, markeer dan "noch eens noch oneens".
3. Deze enquête is volledig anoniem. Niemand zal weten of u ervoor heeft gekozen om deel te nemen of wat uw individuele antwoorden zijn.
4. Het voltooien van de enquête is volkomen vrijwillig, maar we hopen dat u van deze gelegenheid gebruik zal maken om het studentenperspectief over dit belangrijke onderwerp visueel te maken.

Een opmerking over uw privacy

Deze enquête is anoniem.
De bewaarde gegevens bevatten geen identiteitsgegevens tenzij u deze bij een vraag hebt ingevuld. Indien u via een toegangscode deelneemt kunnen wij u verzekeren dat deze niet wordt bewaard in combinatie met uw respons maar wel is opgeslagen in een aparte tabel. De tabel met toegangscodes wordt gebruikt om na te kijken of een enquête reeds voor de betreffende toegangscode is ingevuld. Er is geen enkele manier om de codes te koppelen aan uw respons.

Online vragenlijst:
https://uhasselteconomics.eu.qualtrics.com/jfe/form/SV_dh5jpzamtDEZDVP

INFORMED CONSENT

Ik verklaar hierbij op voor mij duidelijke wijze te zijn ingelicht over de aard en methode van het onderzoek, zoals uiteengezet in de uitnodiging voor dit onderzoek.

Ik stem geheel vrijwillig in met deelname aan dit onderzoek. Ik behoud daarbij het recht deze instemming weer in te trekken zonder dat ik daarvoor een reden hoef op te geven. Ik besef dat ik op elk moment mag stoppen met het onderzoek.

Als mijn onderzoeksresultaten worden gebruikt in wetenschappelijke publicaties, of op een andere manier openbaar worden gemaakt, dan zal dit volledig geanonimiseerd gebeuren. Mijn persoonsgegevens worden niet door derden ingezien zonder mijn uitdrukkelijke toestemming.

Als ik meer informatie wil, nu of in de toekomst, dan kan ik me wenden tot prof. dr. Jochen Bergs. Voor eventuele klachten over dit onderzoek kan ik me wenden tot de Commissie Ethiek UHasselt, per adres: Commissie Ethiek, Universiteit Hasselt, Martelarenlaan 42, 3500 Hasselt; CME@uhasselt.be

❍ ik begrijp de bovenstaande tekst en ga akkoord met deelname aan het onderzoek

REEKS 1: Leren over specifieke onderwerpen omtrent patiëntveiligheid

Onderstaand bevragen we 7 domeinen die te maken hebben met patiëntveiligheid. Geef aan in welke mate jij je vertrouwd voelt met wat je geleerd hebt over elk van deze specifieke inhouden. We vragen je om na te denken over je ervaringen, enerzijds binnen de opleiding (tijdens de lessen), anderzijds binnen het werkveld (tijdens stages). Beoordeel beide settingen afzonderlijk.

		helemaal mee eens	enigszins mee eens	noch eens noch oneens	enigszins mee oneens	helemaal niet mee eens	Ik weet het niet	helemaal mee eens	enigszins mee eens	noch eens noch oneens	enigszins mee oneens	helemaal niet mee eens	Ik weet het niet
		… tijdens de lessen						… tijdens stages					
**Klinische veiligheid**<br>*Ik voel me zelfverzekerd met wat ik geleerd heb over …*													
1	handhygiëne	❍	❍	❍	❍	❍	❍	❍	❍	❍	❍	❍	❍
2	infectiepreventie	❍	❍	❍	❍	❍	❍	❍	❍	❍	❍	❍	❍
3	medicatieveiligheid	❍	❍	❍	❍	❍	❍	❍	❍	❍	❍	❍	❍
4	veilige algemene klinische praktijk	❍	❍	❍	❍	❍	❍	❍	❍	❍	❍	❍	❍
**Werken in een teamverband met andere gezondheidszorg professionals**<br>*Ik voel me zelfverzekerd met wat ik geleerd heb over …*													
5	omgaan met conflicten binnen een interprofessioneel team.	❍	❍	❍	❍	❍	❍	❍	❍	❍	❍	❍	❍
6	verdeling van bevoegdheden, leiderschap en besluitvorming.	❍	❍	❍	❍	❍	❍	❍	❍	❍	❍	❍	❍
7	het aanmoedigen van teamleden tot het uiten van bezorgdheden over veiligheidsproblemen, het in vraag stellen, toetsen en het verdedigen ervan, en indien nodig aansprakelijk te zijn om veiligheidsproblemen gepast te benaderen.	❍	❍	❍	❍	❍	❍	❍	❍	❍	❍	❍	❍
**Effectieve communicatie**<br>*Ik voel me zelfverzekerd met wat ik geleerd heb over …*													
8	verbetering van patiëntveiligheid door middel van duidelijke en consistente communicatie met patiënten.	❍	❍	❍	❍	❍	❍	❍	❍	❍	❍	❍	❍
9	verbetering van patiëntveiligheid door middel van effectieve communicatie met andere zorgverleners.	❍	❍	❍	❍	❍	❍	❍	❍	❍	❍	❍	❍
10	effectieve verbale en non-verbale communicatiemogelijkheden om patiëntveiligheidsincidenten te voorkomen.	❍	❍	❍	❍	❍	❍	❍	❍	❍	❍	❍	❍
**Beheer van veiligheidsrisico's**<br>*Ik voel me zelfverzekerd met wat ik geleerd heb over …*													
11	herkennen van routine situaties waarin patiëntveiligheidsincidenten kunnen ontstaan.	❍	❍	❍	❍	❍	❍	❍	❍	❍	❍	❍	❍
12	identificeren en uitvoeren van veiligheidsmaatregelen.	❍	❍	❍	❍	❍	❍	❍	❍	❍	❍	❍	❍
13	anticiperen op en beheren van risicovolle situaties.	❍	❍	❍	❍	❍	❍	❍	❍	❍	❍	❍	❍
**Inzicht in factoren verbonden aan mens en omgeving**<br>*Ik voel me zelfverzekerd met wat ik geleerd heb over …*													
14	de rol van menselijke factoren, zoals vermoeidheid, bij het ontstaan van patiëntveiligheidsincidenten	❍	❍	❍	❍	❍	❍	❍	❍	❍	❍	❍	❍
15	de rol van omgevingsfactoren, zoals work flow, ergonomie en beschikbare middelen, bij het ontstaan van patiëntveiligheidsincidenten	❍	❍	❍	❍	❍	❍	❍	❍	❍	❍	❍	❍
**Herkennen, reageren en vermelden van patiëntveiligheidsincidenten en bijna-incidenten**<br>*Ik voel me zelfverzekerd met wat ik geleerd heb over …*													
16	herkennen van een patiëntveiligheidsincident of bijna-incident	❍	❍	❍	❍	❍	❍	❍	❍	❍	❍	❍	❍
17	vermindering van de schade door de onmiddellijke risico's voor patiënten en andere betrokkenen aan te pakken	❍	❍	❍	❍	❍	❍	❍	❍	❍	❍	❍	❍
**Veiligheidscultuur**<br>*Ik voel me zelfverzekerd met wat ik geleerd heb over …*													
18	het belang van een kritische ingesteldheid en het uiten van bezorgdheden wanneer u dingen ziet die onveilig kunnen zijn	❍	❍	❍	❍	❍	❍	❍	❍	❍	❍	❍	❍
19	het belang van een ondersteunende omgeving die patiënten en zorgverleners aanmoedigt om bezorgdheden in verband met patiëntveiligheid te uiten	❍	❍	❍	❍	❍	❍	❍	❍	❍	❍	❍	❍
20	systeemeigenschappen (bv. aspecten van de organisatie, het management of de werkomgeving, met inbegrip van beleid, middelen, communicatie en andere processen) en systeem falen en hun rol in patiëntveiligheidsincidenten	❍	❍	❍	❍	❍	❍	❍	❍	❍	❍	❍	❍

REEKS 2: Hoe worden bredere kwesties inzake patiëntveiligheid in de opleiding van gezondheidszorg professionals besproken

Reflecteer over je volledige opleiding (zowel theoretische- als praktijklessen, en stages) tijdens het beantwoorden van onderstaande vragen.

		helemaal mee eens	enigszins mee eens	noch eens noch oneens	enigszins mee oneens	helemaal niet mee eens	Ik weet het niet
21	Als student, was de omvang van mijn job (takenpakket) zeer duidelijk voor mij	❍	❍	❍	❍	❍	❍
22	Er is consistentie in de manier waarop patiëntveiligheidsproblemen werden behandeld door verschillende instructeurs/mentoren tijdens stages	❍	❍	❍	❍	❍	❍
23	Ik had voldoende kansen om te leren en in interactie te treden met leden van interdisciplinaire teams	❍	❍	❍	❍	❍	❍
24	Ik heb goed inzicht verworven dat het rapporteren van incidenten en bijna-incidenten kan leiden tot verandering en het terugdringen van patiëntveiligheidsincidenten	❍	❍	❍	❍	❍	❍
25	Patiëntveiligheid was goed geïntegreerd in de volledige opleiding	❍	❍	❍	❍	❍	❍
26	Klinische aspecten van patiëntveiligheid (bv. handhygiëne, patiënten overdracht, medicatieveiligheid) kwamen voldoende aan bod tijdens de opleiding	❍	❍	❍	❍	❍	❍
27	Systeem aspecten van patiëntveiligheid kwamen voldoende aan bod tijdens de opleiding (bv. aspecten omtrent de organisatie, management of de werkomgeving, met inbegrip van beleid, middelen, communicatie en andere processen)	❍	❍	❍	❍	❍	❍

REEKS 3: Hoe comfortabel voel ik me in het spreken over patiëntveiligheid

Reflecteer over volgende stelling vanuit het idee dat jij bent tewerkgesteld als professioneel gezondheidszorgwerker. Geef aan hoe u zich op dit moment voelt als het gaat over:

		helemaal mee eens	enigszins mee eens	noch eens noch oneens	enigszins mee oneens	helemaal niet mee eens	Ik weet het niet
28	Als ik iemand in mijn werkomgeving onveilige zorg zie verlenen, kan ik deze persoon hierover aanspreken.	❍	❍	❍	❍	❍	❍
29	Als ik een ernstige fout maak, vrees ik dat ik hiervoor disciplinaire maatregelen zal moeten ondergaan.	❍	❍	❍	❍	❍	❍
30	Het is moeilijk beslissingen of handelingen van personen met meer autoriteit in vraag te stellen.	❍	❍	❍	❍	❍	❍
31	In klinische instellingen zijn discussies over patiëntveiligheidsincidenten vooral gericht op systeem gerelateerde problemen, in plaats van zich te concentreren op de persoon / personen die het meest verantwoordelijk zijn voor het incident.	❍	❍	❍	❍	❍	❍

REEKS 4: Demografische kenmerken

Uw leeftijd in jaren: ……… jaar
Uw geslacht: ❍ man / ❍ vrouw / ❍ X

Welke opleiding volgt u momenteel? ❍ verpleegkunde / ❍ geneeskunde / ❍ kinesitherapie
Aan welke school of universiteit volgt u deze opleiding? ❍ PXL / ❍ UCLL / ❍ UHasselt
In welk opleidingsjaar neemt u momenteel de meeste studiepunten op: ❍ 1e bachelor / ❍ 2e bachelor / ❍ 3e bachelor / ❍ 4e bachelor / ❍ 1e master / ❍ 2e master / ❍ 3e master

Heeft u een buitenlandse stage gedaan tijdens uw opleiding?: ❍ ja / ❍ nee
Zo ja, waar (ziekenhuis en land): ……

Heeft u nog bijkomende opmerkingen over patiëntveiligheid tijdens uw opleiding?

………
………
………
………
………
………
………
………
………
………
………
………
………
………
